# Prophage protein RacR activates lysozyme LysN, causing the growth defect of *E. coli* JM83

**DOI:** 10.1038/s41598-019-48690-4

**Published:** 2019-08-29

**Authors:** Qiongwei Tang, Meilin Feng, Bingbing Hou, Jiang Ye, Haizhen Wu, Huizhan Zhang

**Affiliations:** 1 State Key Laboratory of Bioreactor Engineering, East China University of Science and Technology, Shanghai, China; 2 Department of Applied Biology, East China University of Science and Technology, Shanghai, China

**Keywords:** Bacteria, Transcriptional regulatory elements

## Abstract

Prophage enriched the prokaryotic genome, and their transcriptional factors improved the protein expression network of the host. In this study, we uncovered a new prophage-prophage interaction in *E. coli* JM83. The Rac prophage protein RacR (GenBank accession no. AVI55875.1) directly activated the transcription of φ80*dlacZ*Δ*M15* prophage lysozyme encoding gene 19 (GenBank accession no. ACB02445.1, renamed it *lysN*, lysozyme nineteen), resulting in the growth defect of JM83. This phenomenon also occurred in DH5α, but not in BL21(DE3) and MG1655 due to the genotype differences. However, deletion of *lysN* could not completely rescued JM83 from the growth arrest, indicating that RacR may regulate other related targets. In addition, passivation of RacR regulation was found in the late period of growth of JM83, and it was transmissible to daughter cells. Altogether, our study revealed part of RacR regulatory network, which suggested some advanced genetic strategies in bacteria.

## Introduction

Generally, the genotypic diversity in different strains is mainly through mutation, rearrangement or horizontal gene transfer (HGT)^1,2^ and the latter plays a vital role^3,4^. In prokaryote, the pan-genome is the set of all genes^5^, which consists of two parts. The core genome exists in all related bacteria, while only a few bacteria contain the accessory genome^6^, which is acquired via HGT in most cases. Amounts of studies suggest that horizontally acquired DNA (known as mobile genetic elements) comprises transposons, plasmids, and prophages^7,8^, in which prophages are considered as the most significant factor. In the lifecycle of bacteria, some genes encoded by prophage are active and tightly associated with antibiotic resistance, virulence, or metabolism of the host^9^.

*E. coli* diverged from *Salmonella* 100 million years ago^10^, and the K-12 strain acquired nine defective prophages subsequently, including Rac, which was inserted over 4.5 million years ago^11^. The integration or excision of this lambda-like prophage has a great influence on the host^12^. Rac prophage conserves 40% of its original genes that are indispensable for K-12 in specific conditions. φ80 is also derived from lambdoid phage, and a large number of genes maintain the original function^13^. Its derivative φ80*dlacZ*Δ*M15* is generated by aberrant excision and recombination during the integration of φ80^14^. Given their kinship, φ80*dlacZ*Δ*M15* and Rac may be functionally linked, and this connection possibly be due to prophage-prophage interaction.

So far, a large number of studies have focused on the prophage-bacteria regulation, and a few cases in self-regulation of prophages were also reported. At specific time phase or environment, prophages regulators, either proteins or sRNAs, were expressed to control the behavior of the host. As currently reported, phage phi3T-encoded AimR activated the expression of its collinear gene *aimX*, which led to lysis of *Bacillus* cells^15^. And in *E. coli* O157: H7, a prophage CP-933H-encoded regulatory protein, PatE, upregulated the transcription of genes associated with acid resistance and also downregulated the expression of genes that belong to heat shock protein family and type III secretion pathways^16^. The third case is about sRNA DicF, which encoded by Qin prophage. DicF was confirmed to bind to *ftsZ*, *xylR*, and *pykA* mRNAs by directly base pairing and repress their translations. Hence, the cell division and metabolism of bacteria were significantly inhibited^17^.

In the process of transcription, cofactors are indispensable in some networks^18,19^. They usually bind directly to regulatory proteins or the promoter sequence, aimed to enhance binding stability^20^. While in other cases, the transcription can be switched on only when cofactors are modified or disappear because they exert the function as an inhibitor^19,21^. Once the concentration of transcription factors changes, the mRNA levels of target genes would be up-regulated or down-regulated immediately^22^. Occasionally, when the dynamic changes of transcription factors are unable to reserve the overreaction of cells in time, some drastic and irreversible pathways will be activated: (i) the methylation/semi-methylation of promoters can directly block downstream genes^23^. (ii) sRNA can degrade the formed mRNA rapidly, meanwhile, interrupt the extension of the polypeptide^24^. (iii) proteases can destroy the activity of effector proteins. In addition, mRNA methylation has also been revealed, which will alter the half-life of mRNA, or prevent the binding of ribosome^25^. In general, the flexible regulation of gene network has effect on the growth of cell anytime.

RacR is a Rac prophage protein and predicted as a transcriptional factor, which contains a conserved helix-turn-helix (HTH) motif. In this study, we reveal that RacR unexpectedly activates the expression of a distant gene, *lysN*, which is located in φ80*dlacZ*Δ*M15* prophage, and lyses the JM83 cells eventually.

## Results

### Overexpression of RacR induces cell lysis

**Figure 1:**
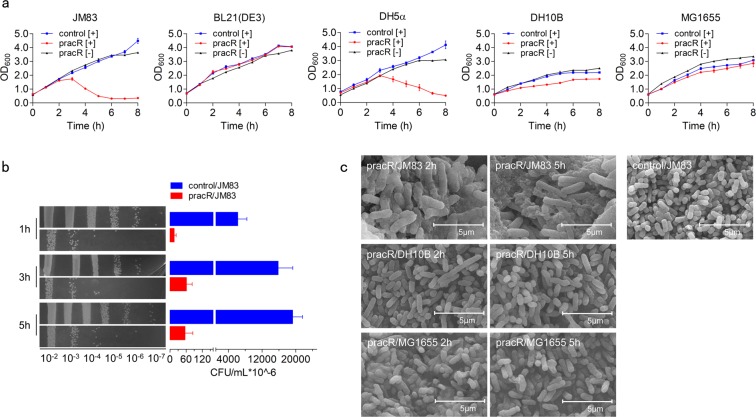
RacR overexpression resulted in JM83 cells lysis. (**a**) Growth curves of JM83, BL21(DE3), DH5α, DH10B, and MG1655 transformed with pBAD or pBAD-racR (pracR) after induction (control [+] in blue and pracR[+] in red) and pracR without induction (pracR [−] in dark). (**b**) Living cell count of JM83 strain transformed with pBAD (control), pracR. 1 h, 3 h, and 5 h represent the time after induction. (**c**) SEM results of the morphology of cells after induction for 2 h or 5 h. Scale bar 5.0 µm. Data represent means ± standard deviations of results from three independent experiments.

We found the growth of JM83 was significantly inhibited by overexpression of RacR, which was also observed in the case of DH5α. However, BL21(DE3) and MG1655 was not affected (Fig. 1a). Accordingly, it seems that the growth inhibition is not caused by toxicity of the protein but has possible genomic context connection. Viability test also suggests a lethal effect of RacR on JM83 (Fig. 1b), which could be further confirmed by serious cell debris in scanning electron microscope (SEM) results. While other strains like pBAD/JM83, pracR/DH10B, pracR/MG1655 seemed normal (Fig. 1c), and the pBAD/JM83 should be regarded as wild-type *E. coli*^26^. Given that RacR belongs to Rac prophage, which does not directly damage cells, we tested the phage bacteriolytic reaction by inoculating the culture of lytic JM83 strain into wild-type *E. coli* at the logarithmic stage, and we found that its growth was completely unaffected. Furthermore, there were no bacteriophage plaques on the LB plate. Altogether, it suggests that RacR probably leads to cell lysis by genome regulation.

### The lysozyme protein LysN in φ80*dlacZ*Δ*M15* is one of the potential targets of RacR

**Figure 2:**
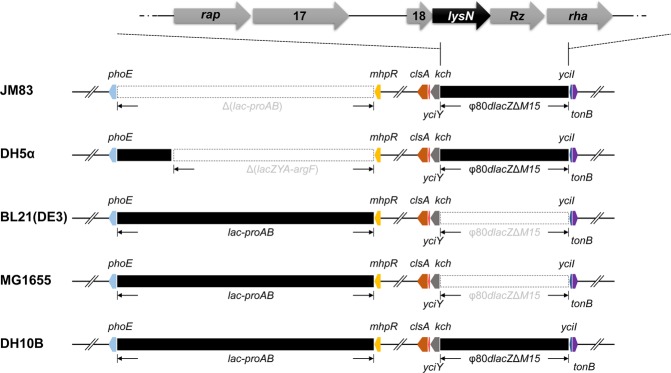
Comparative genomics determined genotype differences. Schematic representation of genotype difference among several *E. coli* strains, mainly depicting two regions, *lac-proAB* and φ80*dlacZ*Δ*M15*. The upper part roughly depicts the gene sites in φ80*dlacZ*Δ*M15*.

To ascertain the downstream regulatory target of RacR, we performed comparative genomics of the above five strains. Interestingly, we found that different with BL21(DE3) or MG1655, JM83 and DH5α both contain φ80*dlacZ*Δ*M15* prophage but lack *lacZYA-argF* gene cluster that harbors CP4-6 prophage (Fig. 2), which may be responsible for lysis phenomenon of these two strains. In addition, overexpression of RacR in DH10B slightly inhibited cell growth (Fig. 1a), seems that *lacZYA-argF* (or maybe CP4-6) is a φ80*dlacZ*Δ*M15* antagonistic cluster.

**Figure 3:**
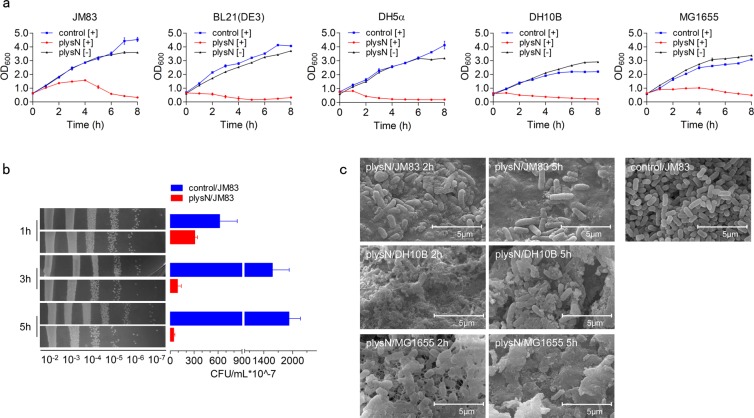
The lysozyme gene *lysN* is a potential target of RacR. (**a**) Growth curves of JM83, BL21(DE3), DH5α, DH10B, and MG1655 transformed with pBAD or plysN after induction (control [+] in blue and pracR[+] in red) and pracR without induction (pracR [−] in dark). (**b**) Living cell count after induction. (**c**) SEM results of the morphology of control/JM83, plysN/JM83, plysN/DH10B, and plysN/MG1655 after induction. Scale bar 5.0 µm. Data represent means ± standard deviations of results from three independent experiments.

Recently, Revathy Krishnamurthi *et al*. uncover some possible binding motifs of RacR, like “GCCTAA” and “TTAGGC”, which lie in the region upstream of *ydaS*^27^. By genome research, we found four potential target genes, 19 (renamed *lysN* in this study), *cI*, *cII*, and 30 (*cro*) in φ80*dlacZ*Δ*M15* contain these motifs (Table 1). Coincidentally, the candidate *lysN* (the black highlighted gene of φ80*dlacZ*Δ*M15*, shown on the top of Fig. 2) is annotated as a lysozyme gene, which is highly probable to participate in the process of cells lysis. Subsequently, LysN was overexpressed via pBAD-lysN (plysN) in JM83. The growth curve showed that the turbidity of culture decreased significantly, while the number of living cells only decreased slightly (Fig. 3a,b). In addition, LysN can also lead to lysis of the other four strains (Fig. 3a), suggesting that LysN may be indeed the direct functional protein associated with lysis. As expected, the fractal pattern due to LysN overexpression observed in JM83, DH10B, MG1655 cells was quite similar to pracR/JM83 overexpression case (Figs 1c and 3c). Combined together, we hypothesize that LysN is one of the direct effectors in the RacR regulatory loop to damage the cells.

**Table 1:** The potential targets of RacR. Bold and underlining, potential binding motifs of each gene.

Gene	Sequence (5′-3′)	Description
***lysN***	GCT**GCCTA**TGGCGCTTCAGCCGGGAGCAT**CCTAA**ACGGCATGTTGAAT**GCCTA**CAG	lysozyme
**CI**	CGT**GGATT**AAATCAACATTATGGTGATGGAAAA**AATCC**ACAT	repressor protein CI
**CII**	CAA**CCTAA**GGCGGAGCCAGGTCACGACAA**AATCCG**CGA	repressor protein CII
**30**	ATGT**GGATT**TTTTCCATCACCATAATGTTGATTT**AATCC**ACG	regulatory protein 30 (*cro*)

### Inactivation of *lysN* rescues JM83 from lysis

**Figure 4:**
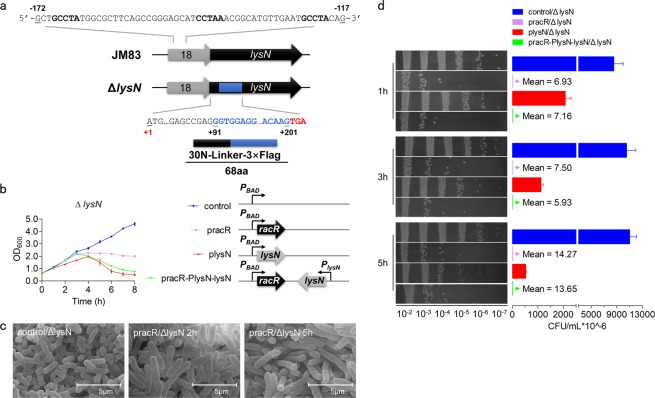
The cell growth was partly recovered after *lysN* mutation. (**a**) Schematic representation of the mutation of *lysN*. The three boldface sequences are predicted binding motifs of RacR. (**b**) Growth curves of Δ*lysN* strain transformed with pBAD, pracR, plysN, and pracR-P_lysN_-lysN after induction. Right part were diagrams of these four plasmids, and *P*_*lysN*_ represents the promoter regions of *lysN*. (**c**) SEM results of the morphology of pracR/Δ*lysN* after induction. Scale bar 5.0 µm. (**d**) Living cell count of Δ*lysN* strains. Data represent means ± standard deviations of results from three independent experiments.

To further confirm whether *lysN* is under control of RacR and accordingly lead to cell lysis, the *lysN* mutant strain was tested, of which the region from +91 to +201 was replaced by a linker plus 3 × Flag-tag (111 bp) and ended with the stop codon TGA (Fig. 4a). Compared to JM83, cell density of *lysN* mutant strain (Δ*lysN*) stayed at a medium level (Fig. 4b), and SEM observation showed that cell debris almost disappeared (Fig. 4c). Taken together, these results showed that inactivation of *lysN* rescues JM83 from lysis. Meanwhile, when introducing the *lysN* expression cassette into pracR, as expected, cells would lyse, which indicated that RacR indeed regulates *lysN in vivo* (Fig. 4b and Supplementary Fig. S1).

We also noticed the cell elongation phenomenon by RacR overexpression in wild type, Δ*lysN* strains via SEM, no matter with or without *lysN* expression (Figs 1c and 4c). This suggests that there may also exist targets other than *lysN* regulated by RacR, which is responsible for cell elongation instead of cell lysis. While although the elongated cells overexpressing RacR didn’t show significant culture turbidity decrease (purple line in Fig. 4b) or cell debris (Fig. 4c) in the first 2–5 hours, the living cell number was decreased significantly (purple bars in Fig. 4d). The decrease of living cells overexpressing RacR was even more serious than *lysN* ovexpressing cells in the first 3 hours but ultimately became close after 5 hours. This may imply that the other regulatory pathway other than *lysN* by RacR is also quite important for cell survival.

### RacR triggers transcription of the *lysN* directly

**Figure 5:**
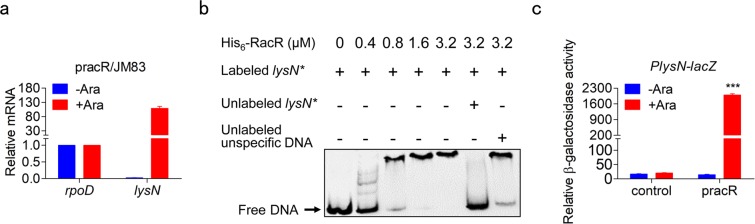
RacR directly activated the expression of *lysN*. (**a**) qRT-PCR analysis of *lysN* in JM83 with pracR. (**b**) EMSA analysis of His_6_-RacR and lysN*. The 150-fold unlabeled lysN* or unlabeled unspecific DNA was added as a control. (**b**) Relative activity of β-galactosidase in Δ*lysN* strain with the LacZ reporter plasmids. Data represent means ± standard deviations of results from three independent experiments. ***P < 0.001.

We then measured the transcription level of *lysN* in pracR/JM83. The accumulation of RacR led to a more than 4000-fold increase in *lysN* mRNA level, which is almost silent without RacR induced expression (Fig. 5a). It suggests that RacR has the ability to trigger the transcription of *lysN*. Then, electrophoretic mobility shift assay (EMSA) was performed with purified His_6_-RacR and the DNA probe (designated lysN^*^: from −235 to +7 relative to start codon of *lysN*). The top of Fig. 4a showed the potential binding motifs in lysN^*^ that derived from half of the palindromic sequence 5′-GCCTAA-3′ and 5′-TTAGGC-3′^27^. As shown in Fig. 5b, His_6_-RacR was observed to bind to lysN^*^ probe in a concentration-dependent manner. This interaction was nearly completely blocked by addition of 150-fold unlabeled lysN^*^, while not by addition of the 150-fold unlabeled unspecific DNA.

Furthermore, the upstream region and 90 bp sequence at the 5′ end of *lysN* was fused to the reporter gene *lacZ*, and the mutations were introduced into the potential binding motifs, as shown in Supplementary Fig. S2. The result showed the activity of controls were extremely low, while overexpression of RacR led to a 140-fold increase (Fig. 5c), which was highly consistent with the transcriptional levels of *lysN* in the genome (Fig. 5a). In addition, base substitution mutation significantly decreased the activity of LacZ, although not completely inhibited (Supplementary Fig. S2). These results demonstrated that RacR activates transcription of *lysN* by directly binding to the promoter region.

### JM83 restored after 18 h of induction due to passivation of regulation

**Figure 6:**
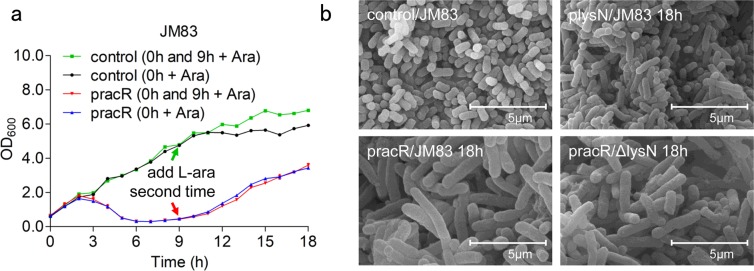
JM83 restored after 18 h of induction. (**a**) Growth curves of JM83 strain transformed with pBAD or pracR after induction. The “0 h + Ara” strains were only added with 0.2% L-arabinose when OD_600_ reached 0.6, and the “0 h and 9 h + Ara” strains were added with enough L-Arabinose again at 9 h after induction (the arrows in green and red represent the time to add L-Arabinose). (**b**) SEM results of four strains after induction. Scale bar 5.0 µm. Data represent means ± standard deviations of results from three independent experiments.

However, we were soon puzzled by the subsequent behavior of pracR/JM83, since the cell density of culture would begin to recover after 8 h of induction (Fig. 6a). What’s more, replenishment of strong inducer at 9 h point would not interfere with the original growth trend (Fig. 6a), indicated that the recovery of growth was not due to insufficiently induced expression of RacR. We then checked the morphology of pracR/JM83, plysN/JM83, and pracR/Δ*lysN* after 18 h of induction, as we expected, Fig. 6b confirmed the recovery of all these strains.

**Figure 7:**
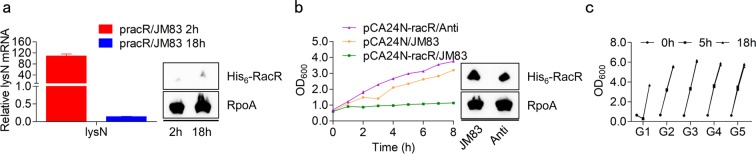
The regulation of RacR is invalid in all daughter cells. (**a**,**b**) qRT-PCR analysis of pracR/JM83 and growth curves of JM83 and Anti after induction. Western blotting indicates the His_6_-RacR level in each strain, and the full-length blot is presented in Supplementary Fig. S4. The Anti strain was depicted in this study. (**c**) The continuous cell culture of pracR/JM83. The whole process contains five generations (G1, G2, G3, G4, and G5). Data represent means ± standard deviations of results from three independent experiments.

To answer this question, we performed qRT-PCR. We found that the mRNA level of *lysN* decreased significantly at 18 h after induction (Fig. 7a). Hence, we inferred that there is a strong connection between *lysN* mRNA level and cell status. We assumed the possibility that the decline of RacR or the passivation of its regulation causes a lower transcriptional level of *lysN*, and detected cellular RacR protein level of each time points. As shown in Fig. 7a, the His_6_-RacR protein level did not change by culture, which excluded the possibility of cascaded decrease of RacR and LysN. Even so, we decided to introduce another plasmid in those recovered cells to produce RacR, in case there are any undetectable changes in pracR. We selected monoclonal recovered pracR/JM83 and renamed it JM83-Anti (resist the overexpression of RacR, Anti for short). After checked the availability in JM83 (Fig. 7b), pCA24N-racR was selected to produce pCA24N-racR/Anti (contain pracR and pCA24N-racR). We did not observe a obvious difference in the expression levels of RacR between JM83 and Anti strain, however, the latter cell growth was no longer inhibited (Fig. 7b). This result strengthened the hypothesis of passivation of RacR regulatory, and suggested this negative effect is permanent. Indeed, continuous cells culture imply the heritable peculiarity of passivation, since all daughter cells grew normally in the presence of L-Arabinose (Fig. 7c, G2 to G5). To sum up, although this particular third factor was not identified, we have uncovered the mechanism of cell growth recovery. In the following research, we will explore more deeply about the passivation of RacR regulatory.

## Discussion

RacR was previously predicted as a transcriptional repressor that belongs to MerR superfamily, whose inhibitory effect on Rac prophage toxin YdaS has been demonstrated recently^27^. In our study, RacR overexpression triggered φ80 lysozyme LysN and resulted in cell lysis. This kind of relationship is highly similar to the effect of RerR on the toxin genes in *Clostridium difficile*^28^. The phage ΦCD119 regulator RerR has been shown inhibit to the distant toxin genes *tcdA* and *tcdR* by directly binding of their promoter, and the PaLoc (pathogenicity locus) of these toxin genes is commonly thought to belong to mobile genetic elements^29^.

The activation and inhibition to LysN and YdaS mean that the host must provide a tight mechanism to adjust the concentration of RacR. In fact, the irregular palindrome in the *racR-ydaS* intergenic region raised the possibility that RacR might negatively regulate its own transcription (Supplementary Fig. S2). This assumption is consistent with the characteristic of regulators of MerR superfamily, which bind to palindrome sequences and were reported to regulate their own expression^30^. Another characteristic of these proteins is the N-terminal located HTH domain and irregular C-terminal domain, the latter is commonly used to bind metal ions, such as Hg^2+^ for Tn501 protein MerR^31^. The *lysN* transcription decreased significantly at 18 h, even reached the same level as the wild-type JM83, while the RacR was constant (Figs 5a and 7a). We propose a hypothesis that the N-terminal of RacR binding the promotor of *lysN* at the early stage, with the assistance of an unknown metal ion. While at the late stage, the concentration of these ions decreased and the free RacR no longer activates the expression of *lysN*. However, only three imperfect conserved motifs were found in the upstream region of *lysN*, and their base composition and spatial arrangement neither can be compared to the regulatory sequence of *ydaS*. More than that, obvious enzyme activity was detected when *lacZ* under the control of a mutant *lysN* promoter (Supplementary Fig. S2), which suggested that these “CCTA” containing motifs were not the key sequences for RacR regulation, and the promoter region of *lysN* contain some more important motifs.

Although we have uncovered the causes of RacR overexpression leading to JM83 lysis, the growth of Δ*lysN* strain cannot recover completely (Fig. 4b), indicated that RacR may also influence other genes. In the early stages of our research, the famous phage regulators CI, CII, and Cro were also considered as potential targets for RacR. We detected their mRNA in Δ*lysN* after 2 h and 18 h of RacR overexpression, as shown in Supplementary Fig. S3. It seems that RacR tried to break the balance of these three regulators in *E. coli* to establish a phage lytic state^32^, since the concentration of primary repressor CI was reduced and its negative regulator Cro was increased. However, we did not find active phages in our solid or liquid medium, and the cultures containing broken JM83 cells were not infectious. We speculate that the defects of φ80*dlacZ*Δ*M15* prophage prevent the assembly and packaging of φ80, but whether CII or Cro plays a role in *lysN* expression and growth defect of Δ*lysN* is unknown. On the other hand, the elongation of cells is probably related to division genes, as the proteins that constitute the divisome^33^, the ZapA-ZapB complex^34^, and the Tol-Pal system^35^. It has been reported that the interference with cell division leads to an elongation phenotype in *E. coli*^36,37^, which is extremely similar to the cell morphology of RacR overexpressing JM83 (Figs 1c and 4c).

In summary, the regulation of RacR to *lysN* is special, since they belong to two different prophages. While in the typical phage lytic cycle, the S holin and R transglycosylase are under the strictly controlled of their own major phage regulators, which activated in the late stage to release the phage^38^. Although the physiological significances for lysozyme activated by foreign regulator still unclear, the model of the cell response to LysN and eventual recovery (Fig. 6a) reveals a diversity of bacterial genetic strategies. We attempted to find clues of the temporal expression of LysN, by introducing a Flag-tag in JM83 genome (Fig. 4a). However, the target protein (LysN_30N-Flag, 8.2 kDa) was not detected successfully, and we supposed that the short half-life period of a small artificial protein affects its detection, since the mRNA of LysN_30N-Flag was transcribed (Supplementary Fig. S5).

## Materials and Methods

### Bacterial strains and growth conditions

Bacterial strains and the plasmids used in this study are listed in Supplementary Table S1. Unless indicated otherwise, *E. coli* were grown aerobically at 37 °C in liquid or on solid Luria-Bertani (LB) media. To better observe the lysis process, the *E. coli* strains harboring pBAD, pCA24N plasmid and their derivatives were cultured at 28 °C after induction. The PCR products of primer pair RacR-1/RacR-2, 19-1/19-2, and RacR-fwd/RacR-rev were ligated into the NdeI/HindIII site of mCherry-pBAD and pCA24N respectively, to forming the pracR, plysN, and pCA24N-racR plasmids. After constructed pracR, the (P19-19)-1/(P19-19)-2 primer pair is used to amplify the *lysN* expression cassette, which ligated into the NdeI site of pracR to yield pracR-PlysN-lysN plasmid. Antibiotics were added at the following concentrations: kanamycin (50 μg/mL), chloramphenicol (30 μg/mL) and/or ampicillin (100 μg/mL) as appropriate.

### Growth curves and spotting assay

Growth was monitored by measuring the optical density (OD) at 600 nm (OD_600_). A single colony of each strain was inoculated in LB and grown at 37 °C overnight. Then, the strains were transferred to 500 mL flasks containing 100 mL of LB medium and were cultured at 37 °C in a shaking incubator (190 rpm). 0.2% L-Arabinose and/or 1 mM IPTG was added when OD_600_ reached about 0.6. We recorded the optical density of these strains at an hour intervals for 8 hours at 28 °C. Meanwhile, 100 μL bacterial suspensions after 1 hour, 3 hours and 5 hours of induction were harvested, 10-fold gradient diluted in fresh LB medium and spread on LB agar plates. The plates were incubated at 28 °C for 24 hours followed by calculating the average colony-forming units (CFU) per milliliter according to the formula [(viable count from each concentration × dilution fold × 10)/n]. Above assays were repeated in triplicate.

### Scanning electron microscope (SEM)

Equivalent cell densities of different *E. coli* strains were collected through centrifugation (2300 × g for 5 min at 4 °C) and washed three times with phosphate buffer (PBS, 0.1 M, pH 7.5). Then, the cell pellets were fixed with 2.5% glutaraldehyde at 4 °C for 5 hours. After washing three times at 4 °C, these samples were dehydrated for 10 min each in increasing concentrations of ethanol (30%, 50%, 70%, 80%, and 90% (V/V)). Subsequently, the samples were frozen at −80 °C for about 24 hours, dried with a vacuum freeze dryer, and then observed with S-3400N scanning electron microscopy.

### Construction of *lysN* in-frame deletion mutant ∆*lysN*

All primers used in mutant construction are listed in Supplementary Table S2. PCR amplifications were performed to generate the upstream fragment of *lysN* with primer pair 19SY-1/19SY-2 and the downstream part with primer pair 19XY-1/19XY-2. Otherwise, we introduced a linker plus 3 × Flag-tag sequences which replaced the in-frame deletion region from +91 to +201 in *lysN* (Fig. 4a). The PCR product containing a site-directed deletion of *lysN* was obtained via overlap PCR with primer pair 19SY-1/19XY-2 and ligated into the NheI/XbaI site of the suicide vector pDMKE (the *insB* deleted derivative of pDMK^39^). The resulting plasmid, pDMKE-*lysN*, were duplicated in *E. coli* DH5α(λpir) and then electrotransformed into *E. coli* JM83. Single colonies selected on LB plate with kanamycin and chloramphenicol suggest that the plasmid was integrated into the chromosome by homologous recombination. The double-crossover recombination was selected on LB plate with 10% sucrose. The *lysN* in-frame deletion mutant was designated as ∆*lysN* and confirmed via PCR and sequencing.

### Quantitative real-time PCR (qRT-PCR)

RNA from *E. coli* JM83 or ∆*lysN* strains frozen at −80 °C was extracted using Pure RNA Isolation Kit according to the manufacturer’s protocols. For removal of the remaining DNA, total RNA was incubated with RNase-free DNase I at 28 °C for 1 hour. 1 μg total RNA was used to generate cDNA using Reverse Transcription M-MLV (RNase H-) kit. Subsequently, quantitative real-time PCR was performed according to SYBR Green PCR Master Mix and each sample was made in triplicate. *rpoD* acts as the internal reference gene. To normalize data, transcription levels of the *rpoD* gene in all samples were set to 1.0. Relative mRNA levels were analyzed using the 2^−ΔCt^ (ΔCt = Ct_*tested genes*_ − Ct _*rpoD*_) method. The primers for qRT-PCR are listed in Supplementary Table S2.

### Overexpression and purification of RacR protein

The *racR* gene was PCR-amplified from *E. coli* JM83 and cloned into the NdeI/EcoRI site of the pET-28a (+) vector to yield pET28a-racR with an N-terminal His_6_-tag. The RacR expression plasmid was transformed into *E. coli* BL21(DE3). The *E. coli* strain was induced with 1 mM IPTG until OD_600_ reached about 0.6 and grown at 16 °C for 16 hours. Then, the strain was harvested by centrifugation (5900 × g for 5 min at 4 °C) and washed three times with phosphate buffer. Pellets were resuspended to a final concentration of 10 OD/mL, sonicated on ice, and centrifuged at 5900 × g for 5 min at 4 °C. The protein was then purified via nickeliminodiacetic acid–agarose chromatography and desalinated into 1 × binding buffer (10 mM Tris-HCl [pH 8.0], 1 mM EDTA, 0.1 M NaCl, 0.1 mM dithiothreitol, 5% glycerol, and 10 μg/mL bovine serum albumin^27^). Purified protein was analyzed by 12% SDS-PAGE, and the protein concentration was determined by the Bradford assay.

### Electrophoretic mobility shift assay (EMSA)

EMSAs were carried out using the purified His_6_-RacR and PCR-amplified DNA probes. The biotin-labeled probes were obtained by PCR with primer head-biotin in Supplementary Table S2, then purified and quantified. Increasing amounts of RacR were added to the 1 × binding buffer that containing target lysN^*^ probes (5 ng) and 50 μg/mL poly(dI·C), and incubated at 28 °C for 40 min. Samples were run on a 6% polyacrylamide gel in 0.5 × TBE buffer at 130 V for 1 hour, then transferred to a nylon membrane at 380 mA for 55 min, subsequently analyzed using chemiluminescent EMSA kits.

### *β-*galactosidase assays

The target fragment was amplified by PCR with primer pair 19_30Z-fwd/19_30Z-rev, 19_30Z-fwd/19M123-4, and 19M123-3/19_30Z-rev respectively, and cloned into the HindIII/PstI of the LacZ reporter vector pXG^40^. The *E. coli* strains containing resulting plasmid were grown at 37 °C in LB and induced by adding 0.2% L-Arabinose when OD_600_ reached about 0.6. After 5 h of induction, the cells were harvested and disrupted in cold phosphate buffer by sonication. The cell debris was removed after centrifugation. Subsequently, β-Galactosidase activity was measured as described previously^41^.

### Western blotting

The target proteins from *E. coli* strains were separated with 12% SDS-PAGE and transferred to PVDF membranes. The samples were blocked in TBS (2% pH 7.5 Tris HCl, 0.8% NaCl) containing 5% skimmed milk overnight at 4 °C. The membranes were probed with anti-His tag primary antibody (1:2000) at room temperature for 2 hours, washed three times with TBS, then incubated with 1:2000 dilution of horseradish peroxidase-conjugated goat anti-mouse antibody at room temperature for 2 hours. Subsequently, the blot was detected using chemiluminescent with TMB.

## Supplementary information

Supplementary materials

## Data Availability

All data generated or analysed during this study are included in this article (and its Supplementary Information Files).

## References

[1] Juhas M (2009). Genomic islands: tools of bacterial horizontal gene transfer and evolution. FEMS Microbiol Rev.

[2] Thomas CM, Nielsen KM (2005). Mechanisms of, and barriers to, horizontal gene transfer between bacteria. Nat Rev Microbiol.

[3] Gogarten JP, Townsend JP (2005). Horizontal gene transfer, genome innovation and evolution. Nat Rev Microbiol.

[4] Ochman H, Lawrence JG, Groisman EA (2000). Lateral gene transfer and the nature of bacterial innovation. Nature.

[5] Mira A, Martín-Cuadrado AB, D’Auria G, Rodríguez-Valera F (2010). The bacterial pan-genome: a new paradigm in microbiology. Int Microbiol.

[6] Soucy SM, Huang J, Gogarten JP (2015). Horizontal gene transfer: building the web of life. Nat Rev Genet.

[7] Heuer H, Smalla K (2007). Horizontal gene transfer between bacteria. Environ Biosafety Res.

[8] Jackson RW, Vinatzer B, Arnold DL, Dorus S, Murillo J (2011). The influence of the accessory genome on bacterial pathogen evolution. Mobile Genetic Elements.

[9] Liu X (2015). Physiological function of rac prophage during biofilm formation and regulation of rac excision in *Escherichia coli* K-12. Sci Rep.

[10] Lawrence JG, Ochman H (1998). Molecular archaeology of the *Escherichia coli* genome. Proc Natl Acad Sci USA.

[11] Wang X (2010). Cryptic prophages help bacteria cope with adverse environments. Nat Commun.

[12] Casjens S (2003). Prophages and bacterial genomics: what have we learned so far?. Mol Microbiol.

[13] Rotman E, Kouzminova E, Plunkett G, Kuzminov A (2012). Genome of enterobacteriophage Lula/phi80 and insights into its ability to spread in the laboratory environment. J Bacteriol.

[14] Durfee T (2008). The complete genome sequence of *Escherichia coli* DH10B: insights into the biology of a laboratory workhorse. J Bacteriol.

[15] Erez Z (2017). Communication between viruses guides lysis-lysogeny decisions. Nature.

[16] Bender JK (2012). Involvement of PatE, a prophage-encoded AraC-like regulator, in the transcriptional activation of acid resistance pathways of enterohemorrhagic *Escherichia coli* strain EDL933. Appl Environ Microbiol.

[17] Balasubramanian D, Ragunathan PT, Fei J, Vanderpool CK (2016). A prophage-encoded small RNA controls metabolism and cell division in *Escherichia coli*. mSystems.

[18] Reiter F, Wienerroither S, Stark A (2017). Combinatorial function of transcription factors and cofactors. Curr Opin Genet Dev.

[19] Schumacher MA, Chinnam NB, Cuthbert B, Tonthat NK, Whitfill T (2015). Structures of regulatory machinery reveal novel molecular mechanisms controlling *B. subtilis* nitrogen homeostasis. Gene Dev.

[20] Zabidi MA, Stark A (2016). Regulatory enhancer-core-promoter communication via transcription factors and cofactors. Trends Genet.

[21] Bass SH, Yansura DG (2000). Application of the *E. coli trp* promoter. Mol Biotechnol..

[22] Johnson MM, Michelhaugh SK, Bouhamdan M, Schmidt CJ, Bannon MJ (2011). The transcription factor NURR1 exerts concentration-dependent effects on target genes mediating distinct biological processes. Front Neurosci.

[23] Low DA, Casadesús J (2008). Clocks and switches: bacterial gene regulation by DNA adenine methylation. Curr Opin Microbiol.

[24] Feng L (2015). A Qrr noncoding RNA deploys four different regulatory mechanisms to optimize quorum-sensing dynamics. Cell.

[25] Peer E, Rechavi G, Dominissini D (2017). Epitranscriptomics: regulation of mRNA metabolism through modifications. Curr Opin Chem Biol.

[26] Xia H (2017). A *yigP* mutant strain is a small colony variant of *E. coli*, and shows pleiotropic antibiotic resistance. Can J Microbiol.

[27] Krishnamurthi R, Ghosh S, Khedkar S, Seshasayee ASN (2017). Repression of YdaS toxin is mediated by transcriptional repressor RacR in the cryptic rac prophage of *Escherichia coli* K-12. mSphere.

[28] Govind R (2009). Bacteriophage-mediated toxin gene regulation in *Clostridium difficile*. J Virol.

[29] Eklund MW, Poysky FT, Reed SM, Smith CA (1971). Bacteriophage and the toxigenicity of *Clostridium botulinum* type C. Science.

[30] Brown NL, Stoyanov JV, Kidd SP, Hobman JL (2003). The MerR family of transcriptional regulators. FEMS Microbiol Rev.

[31] Brown NL, Pridmore RD, Fritzinger DC (1984). The mercury-resistance genes of transposon Tn501: nucleotide sequence of the *mer* operon and a possible mechanism for mercury detoxification. Biochem Soc Trans.

[32] Court DL, Oppenheim AB, Adhya SL (2007). A new look at bacteriophage lambda genetic networks. J Bacteriol.

[33] Du S, Lutkenhaus J (2017). Assembly and activation of the *Escherichia coli* divisome. Mol Microbiol.

[34] Buss JA, Peters NT, Xiao J, Bernhardt TG (2017). ZapA and ZapB form an FtsZ-independent structure at midcell. Mol Microbiol.

[35] de Boer PA (2010). Advances in understanding *E.coli* cell fission. Curr Opin Microbiol.

[36] Krupka M (2017). *Escherichia coli* FtsA forms lipid-bound minirings that antagonize lateral interactions between FtsZ protofilaments. Nat Commun.

[37] Sánchez-Gorostiaga A (2016). Life without division: physiology of *Escherichia coli* FtsZ-deprived filaments. mBio.

[38] Węgrzyn G, Licznerska K, Węgrzyn A (2012). Phage λ-new insights into regulatory circuits. Adv Virus Res.

[39] Xiao J (2009). Characterization of *Edwardsiella tarda rpoS*: effect on serum resistance, chondroitinase activity, biofilm formation, and autoinducer synthetases expression. Appl Microbiol Biotechnol.

[40] Xia H (2017). EsrE-A *yigP* locus-encoded transcript-is a 3′ UTR sRNA involved in the respiratory chain of *E. coli*. Front Microbiol.

[41] Wang Y, Ye J, Zhang H (2012). Identification of transcriptional regulatory sequences of *yigP* gene in *Escherichia coli*. Wei Sheng Wu Xue Bao.

