# Supplementary material for: Prophage protein RacR activates lysozyme LysN, causing the growth defect of E. coli JM83
Source: Sci Rep. 2019 Aug 29;9:12537. doi: 10.1038/s41598-019-48690-4 (PMC6715736; doi:10.1038/s41598-019-48690-4)
Supplement: Supplementary file 1 — Supplementary materials [file 41598_2019_48690_MOESM1_ESM.pdf]

Prophage protein RacR activates lysozyme LysN, causing the growth defect of *E. coli* JM83

Qiongwei Tang[1], Meilin Feng[1], Bingbing Hou[1], Jiang Ye[1], Haizhen Wu[1,2*], Huizhan Zhang[1,2*]

[1]State Key Laboratory of Bioreactor Engineering, East China University of Science and Technology, Shanghai, China

[2]Department of Applied Biology, East China University of Science and Technology, Shanghai, China

*** Correspondence:**
Haizhen Wu
wuhzh@ecust.edu.cn

Huizhan Zhang
huizhzh@ecust.edu.cn

Strain or plasmid	Genotype and/or description	Source or reference
***E.coli* strains**		
JM83	Wild-type; *rpsL* ara Δ(*lac-proAB*) φ80*dlacZ*Δ*M15*	[1]
DH5α	F^- *endA1 glnV44 thi-1 recA1 relA1 gyrA96 deoR nupG purB20* φ80*dlacZ*Δ*M15* Δ(*lacZYA-argF*)U169, *hsdR17*(r_K^- m_K^+), λ^-	[2]
MG1655	K-12 F^- λ^- *ilvG*$^-$ *rfb-50 rph-1*	[3]
BL21(DE3)	*E. coli* str. B F^- *ompT gal dcm lon hsdS*$_B$(r_B^- m_B^-) λ(DE3 [*lacI lacUV5-T7p07 1 ind1 sam7 nin5*]) [*malB*$^+$]$_{K\text{-}12}$(λ^S)	[4]
DH10B	F^- *endA1 deoR*$^+$ *recA1 galE15 galK16 nupG rpsL Δ(lac)X74* φ80*dlacZ*Δ*M15 araD139* Δ(*ara,leu*)*7697 mcrA* Δ(*mrr-hsdRMS-mcrBC*) Str^R λ^-	[5]
Δ*lysN*	Δ*lysN*:: linker-3 × Flag-tag of JM83	This work
**Plasmids**		
mCherry-pBAD	arabinose inducible vector	Addgene
pBAD-racR	pBAD carrying *racR* gene with N-terminal His_6-tag	This work
pBAD-lysN	pBAD carrying *lysN* with N-terminal His_6-tag	This work
pBAD-racR-P_{lysN}-lysN	pBAD-racR carrying *lysN* expression cassette in reverse orientation	This work
pET28a-racR	pET28a carrying *racR* gene with N-terminal His_6-tag	This work
pDMKE	suicide plasmid used for markless deletions of genes	This work
pDMKE-lysN	pDMKE carrying *lysN*	This work
pCA24N	high copy number plasmid with IPTG-inducible promoter, $P_{T5\text{-}lac}$ and His_6-tag attached to the N-terminal	[6]
pCA24N-racR	pCA24N carrying *racR* gene with N-terminal His_6-tag	This work
pCA24N-lysN	pCA24N carrying *lysN* with N-terminal His_6-tag	This work
pXG-P_{lysN}-lacZ	*lacZ* reporter gene controlled by P_{lysN}	This work
pXG-P_{lysN}M-lacZ	*lacZ* reporter gene controlled by $P_{lysN}M$	This work

Table S1. Strains and plasmids used in this study.

Primers	Sequence (5' to 3')
**Construction of *racR* and *lysN* overexpression plasmids**	
RacR-1	CTAGCTAGCATGCTTAGTGGTAAAGACTTAG
RacR-2	CCCAAGCTTTTAAGTTCCAGTTTTTGTGATG
19-1	ATCATCATGGTATGGAATTCATGAACCCAACGTTGAGG
19-2	CCGCCAAAACAGCCAAAGCTTTCATTTCTGGCCCCACTC
(P19-19)-1	TGTGCGGTATTTCACACCGTATCGCATGGACAAACTAAC
(P19-19)-2	GATTGTACTGAGAGTGCACTCATTTCTGGCCCCACTCGC
RacR-fwd	ATGCTTAGTGGTAAAGACTTAG
RacR-rev	TTAAGTTCCAGTTTTTGTGATG
**Construction of Δ*lysN***	
19SY-1	GGTTACCCGGATCTATCTAGTCATCTCTAACGCTGGGAA
19SY-2	GCCTGAACCGCCTCCACCCGCATTGCCCAGCATTACT
19XY-1	GACGATGACGACAAGTGAGAGTGTGACAACCTGCTTAA
19XY-2	CTAGTGGGGCCCTTCTAGTGCGCGGTTATCCTGTCGA
Flag-1	GTAATGCTGGGCAATGCGGGTGGAGGCGGTTCAGGCG
Flag-2	AAGCAGGTTGTCACACTCTCACTTGTCGTCATCGTCC
Flag-JD-1	CAAGGACCACGACGGCGACTACAAG
Flag-JD	CTTGTCGTCATCGTCCTTGTAGTCG
C1(19)	GGCTGATTCCATTCTCTCAAAAGTA
C2(19)	GCGTTTACTGCGGACATAAAAAAGC
19(Sq)	TCATTTCTGGCCCCACTCGCAGACT
19(prev)-1	ATCAATCAATACAAGGCTGCCGTTC
19(prev)-2	CGACCACATCCTGATAGGCGTAATA
**qRT-PCR**	
19(RT)-JD-1	CAAGGTTCGCATCCCTGAGCC
19(RT)-JD-2	CCCCTTCCACTGCTTGCCGCC
cI(RT)-1	GACTCTGGCGGGATGTTGCT
cI(RT)-2	CGGGATTTGGCTGAGGCTTT
cII(RT)-1	TCGCTTTCTGGATAACCGC
cII(RT)-2	GAGAACGGCTTGCTTTGCT
30(RT)-1	AAAAATCCAACGCCAGCACCC
30(RT)-2	GGCGGCAAAGATACCTGGATA
rpoD-X1	CAACCAGGTTCAATGCTCCGTT
rpoD-X2	TTCCTGGGAAAGCTCAGAACCGA
19_30N-Flag (RT)-1	GCTGGTGGGTGCCATTGTT
19_30N-Flag (RT)-2	CACTTGTCGTCATCGTCCTT
**Probes used in EMSA**	
19-fwd	AGCAACAGCACTCGCACAAA
19-rev	GGTTCATCTCGGCTCCTGCT
head-biotin	AGCCAGTGACGATAAG
**Construction of reporter plasmids in β-galactosidase assays**	
19_30Z-fwd	CCCTTTCGTCTTCACAAGCTTAAATAATTTTCCCTAATG
19_30Z-rev	CGTAATCATGGTCATGCTAGCCGCATTGCCCAGCATTAC
19M123-3	TGGCGCTTCAGCCGGGAGCATAAGCCACGGCATGTTGAATTAAGCCAGCCCCGAGCAGTGGAA
19M123-4	ATGCTCCCGGCTGAAGCGCCAGCTTAAGCACCAGTGGTTAGTTTGTCCAT

Table S2. Primers used in this study.

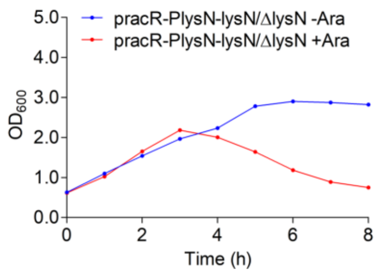

Fig. S1: Growth curves of Δ*lysN* strains transformed with pracR-P_{lysN}-lysN. Data represent means ± standard deviations of results from three independent experiments.

Binding sequences 5'-AATA**TTAGGC**GAC**GCCTAA**CGCAAGTCA**ATAGGC**TGT**GCCTAA**TGCAGTAAGGGTAG**GGATTGCCTAA**TGTAATGCGCATA-3'

***P_{lysN}*_N30** 5'-TGCT**GCCTAT**GGCGCTTCAGCCGGGAGCA**TCCTAA**ACGGCATGTTGAAT**GCCTAC**AGCCCCG...ATG...84bp...CCA-3'

***P_{lysN}*M_N30** 5'-TGCT**TAAGCT**GGCGCTTCAGCCGGGAGCA**TAAGCC**ACGGCATGTTGAAT**TAAGCC**AGCCCCG...ATG...84bp...CCA-3'

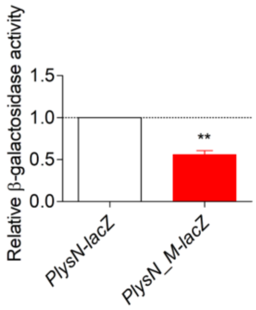

Fig. S2: **Schematic representation of the putative RacR binding region in *P_{lysN}*.** The boldface and underlined sequences were previously reported binding motifs of RacR. *P_{lysN}*_N30 comprised the upstream region and 90 bp sequence of the N-terminal of *lysN*. Mutations were introduced into *P_{lysN}*_N30 to produce *$P_{lysN}M$*_N30. The bottom histogram is the relative activity of β-galactosidase in Δ*lysN* strain with the *lacZ* reporter plasmids. Data represent means ± standard deviations of results from three independent experiments. **, P<0.01.

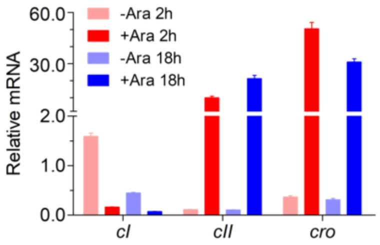

Fig. S3: **qRT-PCR analysis of potential targets in JM83 with pracR.** Relative mRNA levels were analyzed using $2^{-\Delta Ct}$ (ΔCt = $Ct_{tested\ genes}$ - Ct_{rpoD}) method. Data represent means ± standard deviations of results from three independent experiments.

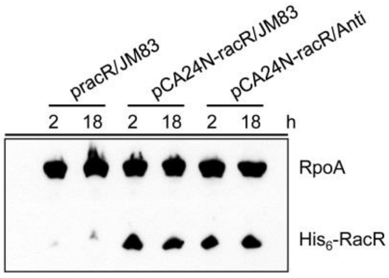

Fig. S4: **Western blotting of RacR overexpression strains**. Columns 1, 2, 3, and 5 are shown in **Figure 7**.

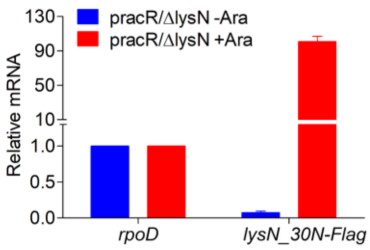

Fig. S5: **qRT-PCR analysis of *lysN*_30N_Flag.** The *rpoD* acts as an internal reference gene. Data represent means ±

standard deviations of results from three independent experiments.

References

1 McLean, K. M., Gutman, P. D., Minton, K. W. & Clark, E. P. Increased resistance to ionizing and ultraviolet radiation in *Escherichia coli* JM83 is associated with a chromosomal rearrangement. *Radiat Res* **130,** 366-371 (1992).

2 Chen, J., Li, Y., Zhang, K. & Wang, H. Whole-genome sequence of phage-resistant strain *Escherichia coli* DH5α. *Genome Announc* **6,** e00097-18 (2018).

3 Hayashi, K. *et al.* Highly accurate genome sequences of *Escherichia coli* K-12 strains MG1655 and W3110. *Mol Syst Biol* **2,** 2006.0007 (2006).

4 Jeong, H. *et al.* Genome sequences of *Escherichia coli* B strains REL606 and BL21(DE3). *J Mol Biol* **394,** 644-652 (2009).

5 Durfee, T. *et al.* The complete genome sequence of *Escherichia coli* DH10B: insights into the biology of a laboratory workhorse. *J Bacteriol* **190,** 2597-2606 (2008).

6 Guo, Y. *et al.* RalR (a DNase) and RalA (a small RNA) form a type I toxin-antitoxin system in *Escherichia coli*. *Nucleic Acids Res* **42,** 6448-6462 (2014).
